# Surface Expression and Subunit Specific Control of Steady Protein Levels by the Kv7.2 Helix A-B Linker

**DOI:** 10.1371/journal.pone.0047263

**Published:** 2012-10-24

**Authors:** Paloma Aivar, Juncal Fernández-Orth, Carolina Gomis-Perez, Araitz Alberdi, Alessandro Alaimo, Manuel S. Rodríguez, Teresa Giraldez, Pablo Miranda, Pilar Areso, Alvaro Villarroel

**Affiliations:** 1 Unidad de Biofísica, CSIC, UPV/EHU, Universidad del País Vasco, Barrio Sarriena s/n, Leioa, Spain; 2 Dept. Farmacología, UPV/EHU, Universidad del País Vasco, Barrio Sarriena s/n, Leioa, Spain; 3 Proteomics Unit, CIC bioGUNE CIBERehd, Technology Park of Bizkaia, Building, Derio, Spain; 4 Unidad de Investigación, Hospital Universitario Ntra Sra Candelaria, Santa Cruz de Tenerife, Spain; Sackler Medical School, Tel Aviv University, Israel

## Abstract

Kv7.2 and Kv7.3 are the main components of the neuronal voltage-dependent M-current, which is a subthreshold potassium conductance that exerts an important control on neuronal excitability. Despite their predominantly intracellular distribution, these channels must reach the plasma membrane in order to control neuronal activity. Thus, we analyzed the amino acid sequence of Kv7.2 to identify intrinsic signals that may control its surface expression. Removal of the interlinker connecting helix A and helix B of the intracellular C-terminus produces a large increase in the number of functional channels at the plasma membrane. Moreover, elimination of this linker increased the steady-state amount of protein, which was not associated with a decrease of protein degradation. The magnitude of this increase was inversely correlated with the number of helix A – helix B linkers present in the tetrameric channel assemblies. In contrast to the remarkable effect on the amount of Kv7.2 protein, removal of the Kv7.2 linker had no detectable impact on the steady-state levels of Kv7.3 protein.

## Introduction

Kv7 (KCNQ) channels are of crucial importance in excitable tissues [1.2]. In the nervous system, Kv7.2 and Kv7.3 are the main components of the M-current, a voltage-dependent, non-inactivating K^+^ current that plays a fundamental role in controlling the activity of both peripheral and central neurons [3]. The number of Kv7 channels at the plasma membrane determines the electrical properties of a cell. Failure to control this number affects cellular excitability, as seen in Benign Familial Neonatal Convulsions (BFNC), a dominantly inherited idiopathic human epilepsy caused by spontaneous mutations in Kv7.2 or Kv7.3 [4].

Although Kv7.2 acts in the plasma membrane, the majority of this protein is located in the ER and other intracellular compartments, both in neurons and in cells heterologously expressing the channel [5.6]. A membrane protein must pass through multiple quality control mechanisms before reaching the plasma surface that begin with the translation of the protein and that involve subunit assembly, ER exit, transport to the membrane and their maintenance therein, endocytosis and degradation [7], [8]. These overlapping events control ion channel expression at the cell surface and may be coordinated or even cooperate [7]. However, the mechanisms that control the abundance of Kv7 channels at the membrane are generally poorly understood. As for other membrane proteins, these mechanisms hinge on intrinsic and extrinsic signals. The reentrant loop that forms the pore is the strongest intrinsic signal known to control the surface expression of M-channels [9]. In conjunction with helix D located in the intracellular C-terminus that constitutes the assembly domain [10]–[12], this region drives the increase in the number of M-channels observed upon heteromerization. To date, calmodulin (CaM) is the only extrinsic physiological signal known to regulate Kv7 channels trafficking [13]. CaM binds to helix A and B of the intracellular C-terminus [14]–[16], and some mutations linked to BFNC that are located in helix A [17], [18] reduce CaM binding, thereby leading to ER retention and the consequent reduction in surface expression [13], [19].

While the mechanisms controlling surface expression of certain membrane proteins are well documented [8], [20], [21], little is known about the intrinsic signals that influence the surface expression of Kv7 channels. Accordingly, we have analyzed the Kv7.2 subunit sequence to identify intrinsic signals that control trafficking, and we found that the linker between helices A and B regulates both surface expression and protein production.

## Results

**Figure 1:**
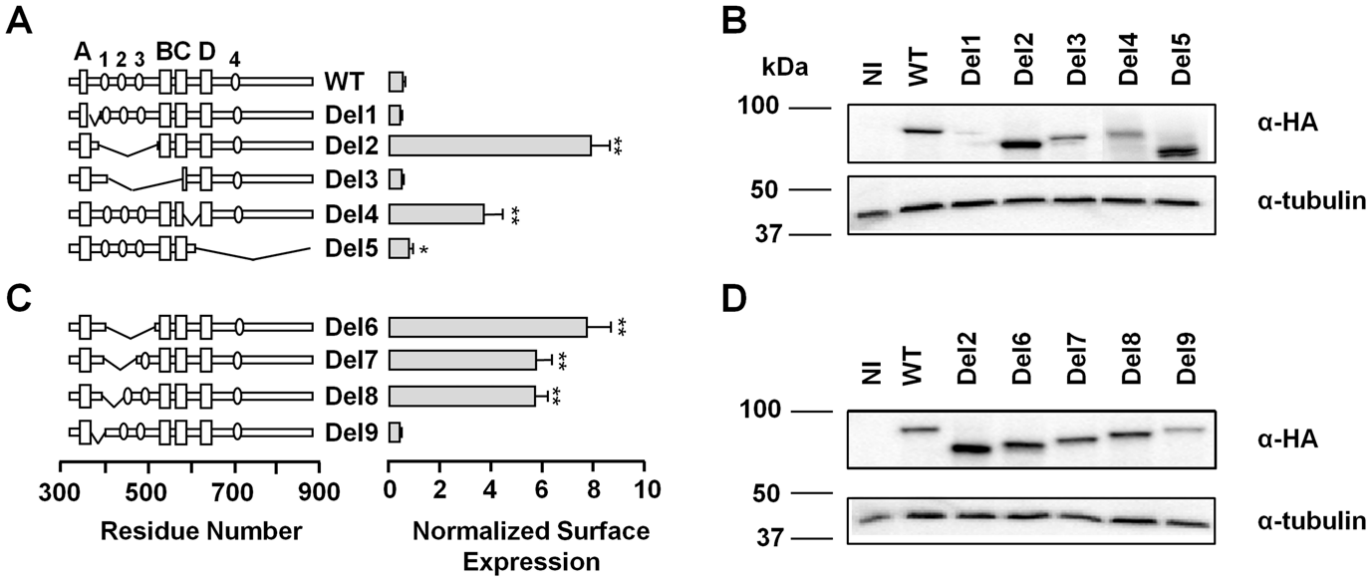
Identification of C-terminal regions that influence Kv7.2 surface expression. *A.- and C.-* Left, schematic representation of serial deletions introduced into the intracellular C-terminal Kv7.2 sequence. The boxes in the expanded intracellular C-terminal scheme indicate regions with a high probability of adopting an alpha helix configuration, and ovals (numbered 1 to 4) indicate regions with a significant PESTfind score. The PESTfind score (www.biu.icnet.uk/projects/pest) was 19.1, 12.6, 6.1, and 8.6 for PEST1 (_397_KDPPPEPSPSQK_408_), PEST2 (_437_RSPSADQSLEDSPS_451_), PEST3 (_476_RQNSEEASLPGEDIVDD_493_), and PEST4 (_803_RPYIAEGESDTDSDLCTPCGPPPR_826_), respectively. Right, normalized surface expression in *Xenopus* oocytes of the Kv7.2 subunits indicated on the left tagged with HA at the extracellular S1–S2 loop (*n* ≥12, two batches). The amount of Kv7.2-HA containing channels in the oocyte membrane was quantified using a single whole-oocyte chemiluminescence assay (see Materials and Methods). The background of uninjected oocytes was subtracted and the values given are the means (± SEM) normalized to the values obtained from WT-Kv7.2-HA channels from the same batch. * *P*≤0.05; *** *P*≤0.001; unpaired Student’s *t* test. ***B.- and D.-*** Steady-state protein levels differed among the various mutant subunits. Proteins from *Xenopus* oocytes injected with the same amount of mRNA expressing the indicated HA-tagged constructs were separated by SDS-PAGE and analyzed in Western blots probed with anti-HA antibodies (*n* = 4).

A systematic evaluation of Kv7.2 C-terminal deletion constructs in *Xenopus* oocytes revealed a remarkable effect of the linker between helices A and B on surface expression (Fig. 1A), which could be measured using an extracellular HA epitope tag located between S1–S2 [10], [11]. In addition to the helix A-B linker, the loop connecting helices C and D exerted an important influence on surface expression (see Del2 ΔT359–T501 and Del4 ΔL548–M591, Fig. 1A). Moreover, truncation after helix C (Del5 ΔS590-stop) augmented surface expression, although not to the extent of the deletions between helices A–B or C–D.

Removal of different regions within the AB linker led to increased surface expression (Fig. 1C) and protein production (Fig. 1D). Del8 (ΔY372-K408) was the smallest deletion analyzed that augmented surface expression, while, Del9 (ΔT359-Y372) had no effect on surface expression or total protein signal. Except for Del9, comparable increases in protein yield were observed for the different AB linker deletion mutants (Figs. 1B and 1D), ranging from 2.35 to 3.30 fold. The average pooled increase in protein yield for Del2, Del6, Del7 and Del8 was 2.73±0.21 fold (*n* = 10). Unlike deletions of the helix A-B loop (Del2 ΔT359–T501, Del6 ΔY372-K493, Del7 ΔY372-S450 and Del8 ΔY372-K408, Figs. 1B and 1D), the steady-state protein levels were not so obviously affected by removal of the linker between helices C and D (Del4; Fig. 1B). As the greatest influence on surface expression was observed for mutants in which the helix A–B linker was removed, we focused our analysis on this region.

**Figure 2:**
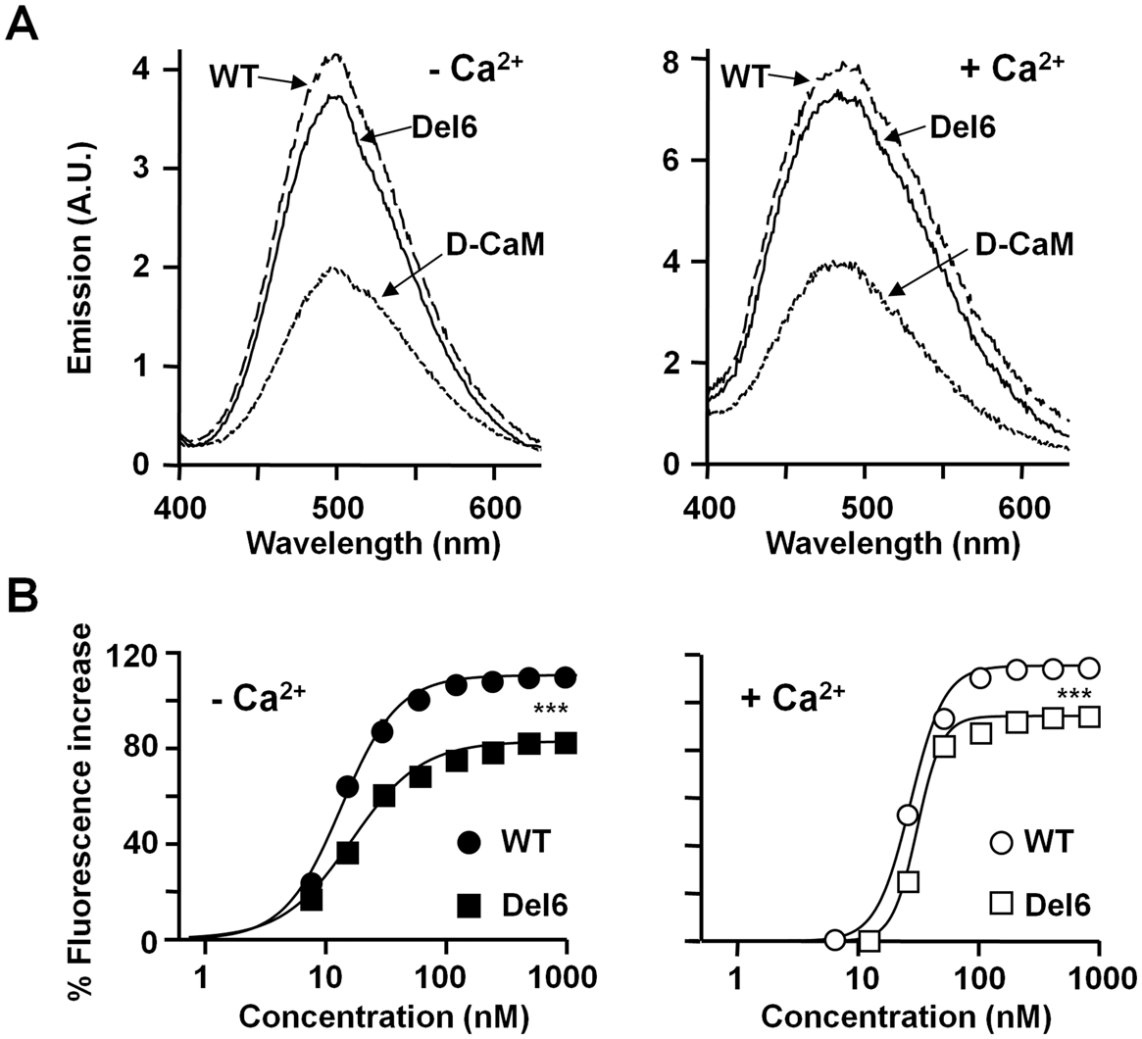
Characterization of the interaction of Del6 with CaM. *A.* Emission spectra of 12.5 nM D-CaM in the absence (left panel; 10 mM EGTA and no added Ca^2+^), and in the presence of 2 µM free Ca^2+^ (right panel), as well as in the presence of molar excess of the indicated GST-Kv7.2 fusion proteins (200 nM). Note the difference in the fluorescence emission axis, as well as the shift in the peak emission to the left in the presence of Ca^2+^. ***B.*** Relative concentration-dependent enhancement of D-CaM fluorescence emission in the absence (left) and presence of 2 µM free Ca^2+^ (right). The parameters used to fit a Hill equation to the data for WT in absence of Ca^2+^ were: Max = 111±1.4, EC_50_ = 11±0.5 nM, h = 1.8±0.2 (*n* = 9). For Del6 in absence of Ca^2+^: Max = 83±1.3, EC_50_ = 13±0.7 nM, h = 1.5±0.1 (*n* = 5). For WT in the presence of Ca^2+^: Max = 115±2.2, EC_50_ = 27±1.2 nM, h = 3.1±0.4 (*n* = 9). For Del6 in presence of Ca^2+^: Max = 95±1.1, EC_50_ = 31±0.8 nM, h = 4.2±0.3 (*n* = 5). The data represent the means ± standard error from five or more independent experiments. The error bars are smaller than the symbols.

The impact of the linker on calmodulin (CaM) binding cannot explain the changes in surface expression. CaM binds to helix A and B [14], [15] regulating the exit of Kv7.2 subunits from the ER [13]. Corroborating our previous findings [14], co-immunoprecipitation demonstrated that CaM interacted with the channels in both the presence and absence of the linker (data not shown). Thus, to examine the interaction in more detail, we performed *in vitro* binding assays using a fluorescent derivate of CaM (D-CaM) that increases its fluorescent emission upon binding to the target [19]. The apparent affinity observed in the dose-response for the CaM-Del6 binding was almost identical to that obtained with the WT binding site, while the maximal fluorescence emission was only slightly but significantly reduced (Fig. 2). In this assay we have already demonstrated a correlation between reduced fluorescence and decreased in surface expression [19]. Therefore, the impact of the linker on surface expression appears to be unrelated to its interaction with CaM.

**Figure 3:**
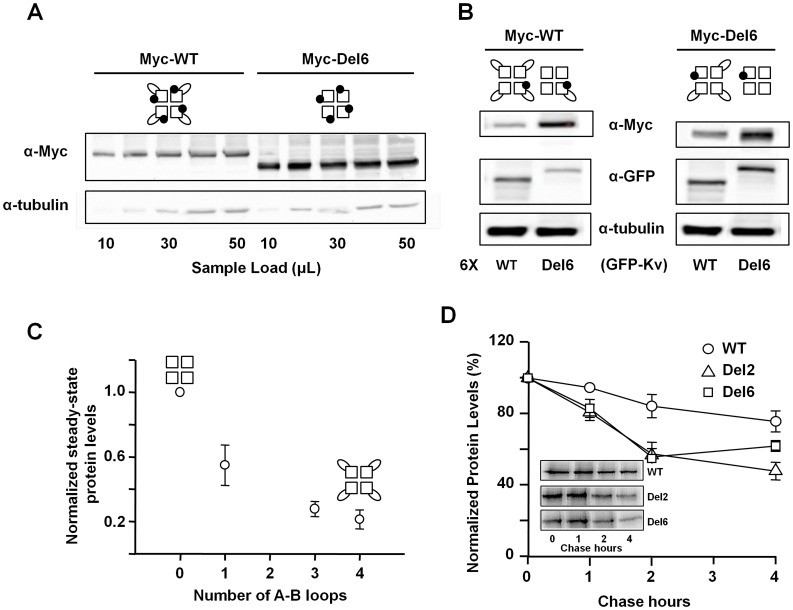
The Del6 mutant subunit presents increased Kv7.2 total protein. *A.-* Western blot of increasing sample load from HEK293T cells extracts expressing the same amount of DNA coding WT- or Del6-Kv7.2 Myc-tagged subunits. The optical densities (OD) of the bands were analyzed using ImageJ software. A linear regression fit was obtained from the OD *vs* load relation. From this regression, the amount required to obtain the same OD’s was found to be 4.7±1.22 fold larger (*n* = 5) for WT subunits than for Del6 mutant Kv7.2 subunits. Top, the cartoons are schematic representations of the tetrameric assemblies. Each square represents a subunit of the tetrameric channel, the ovals represent the A-B loops, and the black circles highlights the presence of a Myc tag. ***B.-*** The steady-state protein levels are inversely proportional to the number of A–B loops present in the tetrameric Kv7.2 assemblies. Western blot of protein extracts from HEK293T cells expressing Del6- (right panel) or WT- Myc-Kv7.2 subunits (left panel) when co-expressed with a six fold larger amount of plasmid DNA coding the indicated YFP-tagged protein (*n* = 4). Myc tagged WT and Del6 Kv7.2 channels were detected using anti-Myc antibody. The Del6 protein levels decreased when co-expressed with WT subunits, whereas increased protein levels of WT Kv7.2 were detected when co-expressed with Del6. A schematic representation of the theoretical assemblies detected with the anti-Myc antibody is represented at the top. The subunits that were overexpressed and that lack a Myc tag, so they are not detected in the Western blot, are indicated at the bottom of each lane. ***C.-*** Normalized quantification of Western blot signals as a function of the theoretical number of A–B loops present on the detected channel assemblies. On each Western blot, the OD’s were normalized to the signal obtained for tubulin. The values were subsequently normalized to those obtained with Myc-Del6 or Myc-WT in the same Western blot. Data points for 0 (Del6) and 4 (WT) loops were derived from data obtained as in panel A, data points for 1 and 3 loops were obtained from Western blots equivalent to the left and right columns in panel B, respectively. The points represent the means ± S.E.M. (n ≥4). ***D.-*** Pulse-chase analysis of WT-, Del2- and Del6-Kv7.2 subunit stability. Densitometric quantification of the bands normalized to the value at time 0 (no chase). Each data point is the mean ± SEM calculated from three separate experiments. Inset: representative images of autoradiographic films of experiments in HEK293T cells transfected with the indicated plasmids. Metabolic labeling was performed for 1 h, 36 h post-transfection, followed by chase times of 1, 2 and 4 h.

In accordance with the results of the oocyte experiments, removal of the A–B linker increased the protein yield in mammalian cells. Protein levels induced by the expression of the WT and Del6 constructs were compared by loading increasing amounts of the sample protein extracts into SDS gels, which were then analyzed in Western blots. To obtain a signal comparable to that seen for the Del6 extracts, approximately fivefold more sample was required from cells expression WT subunits (Fig. 3A).

The number of loops present in the tetrameric Kv7.2 channel was inversely correlated with steady-state protein expression. Indeed, the protein yield in cells expressing loop-less mutant subunits was reduced when they were co-expressed with excess WT subunits (Fig. 3B, right panel). In this experiment, only channels incorporating at least one Myc-tagged subunit could be detected and, due to the greater amount of cDNA encoding non-Myc-tagged WT subunits (6 fold that of the mutant subunits), the vast majority of the channels detected contained of one Myc-tagged mutant subunit and three non-Myc-tagged WT subunits. The rationale is that, given that assembly of Kv7.2 is a random process [22], mass action will favor the assembly of Myc-tagged subunits with the most abundant non-tagged subunits. Thus, these channels contained 3 and 0 loops, respectively, and accordingly, we could plot the relative protein abundance as a function of the number of loops (Fig. 3C). Similarly, the relative protein abundance of channels having 4 and 1 loops was examined (Fig. 3B, left panel), and the results were incorporated into Fig. 3C. Given the increase in protein levels and surface expression observed in oocytes expressing different helix A-B linker deletion mutant constructs, these results suggest a causal relationship between protein yield and surface expression.

The presence of the A-B loop did not augment the rate of protein degradation (Fig. 3D). The increase in protein yield may be due to a reduction in the rate of degradation or, alternatively, to an increase in the rate of protein synthesis. The sequence connecting helices A and B reveals the existence of three PEST motives (ovals in Fig. 1). These motives, which are rich in proline (P), glutamate (E), serine (S) and threonine (T) are often encountered in CaM binding proteins that are susceptible to proteolysis by endogenous neutral proteases such as calpain I and calpain II [23], and have been found to promote the rapid degradation of proteins [24]. Therefore, we tested the impact of the loop on protein degradation. HEK293T cells were treated with the protein synthesis blocker cycloheximide (CHX) for various time periods and the remaining protein levels after degradation were evaluated by Western blotting. However, contrary to our expectations, in four independent experiments we did not observe differences in the stability of the protein that could account for the increased protein yield (not shown). We also tested the effect of lysosomal and proteasomal inhibitors. No significant differences were observed after 10 h treatment with the proteosomal inhibitor MG132 (n = 3), as found by others [6]. After 10 h of treatment with the lysosomal inhibitor chloroquine, the levels of channels carrying the A-B loop tended to be larger (n = 3, see Fig. S1). However, the differences were modest, and further experiments are required to test the contribution of this pathway. We also addressed the impact of the loop on the regulation by the ubiquitin ligase Nedd4-2. Ubiquitination results in the removal of some ion channels, receptors and transporters from the membrane [25], and it has been shown that Kv7.2/3 channels are regulated by Nedd4-2 and ubiquitination [26]. We confirmed that overexpression of Nedd4-2 down-regulates de current mediated by heteromeric Kv7.2/3 channels, but has no effect on the current mediated by homomeric Kv7.2 channels (n >7, not shown). Similarly, Nedd4-2 did not have any significant effect on the current levels of cells expressing homomeric Del6 loop-less mutant channels (n = 11, not shown). To further test the impact of the loop on the stability of Kv7.2, we performed pulse and chase experiments that revealed that the degradation rate was not reduced after loop removal (Fig. 3D). On the contrary, the removal of the loop apparently led to a faster degradation rate. The simplest explanation, therefore, is that the presence of the loop causes a reduction on the rate of synthesis of Kv7.2 proteins.

**Figure 4:**
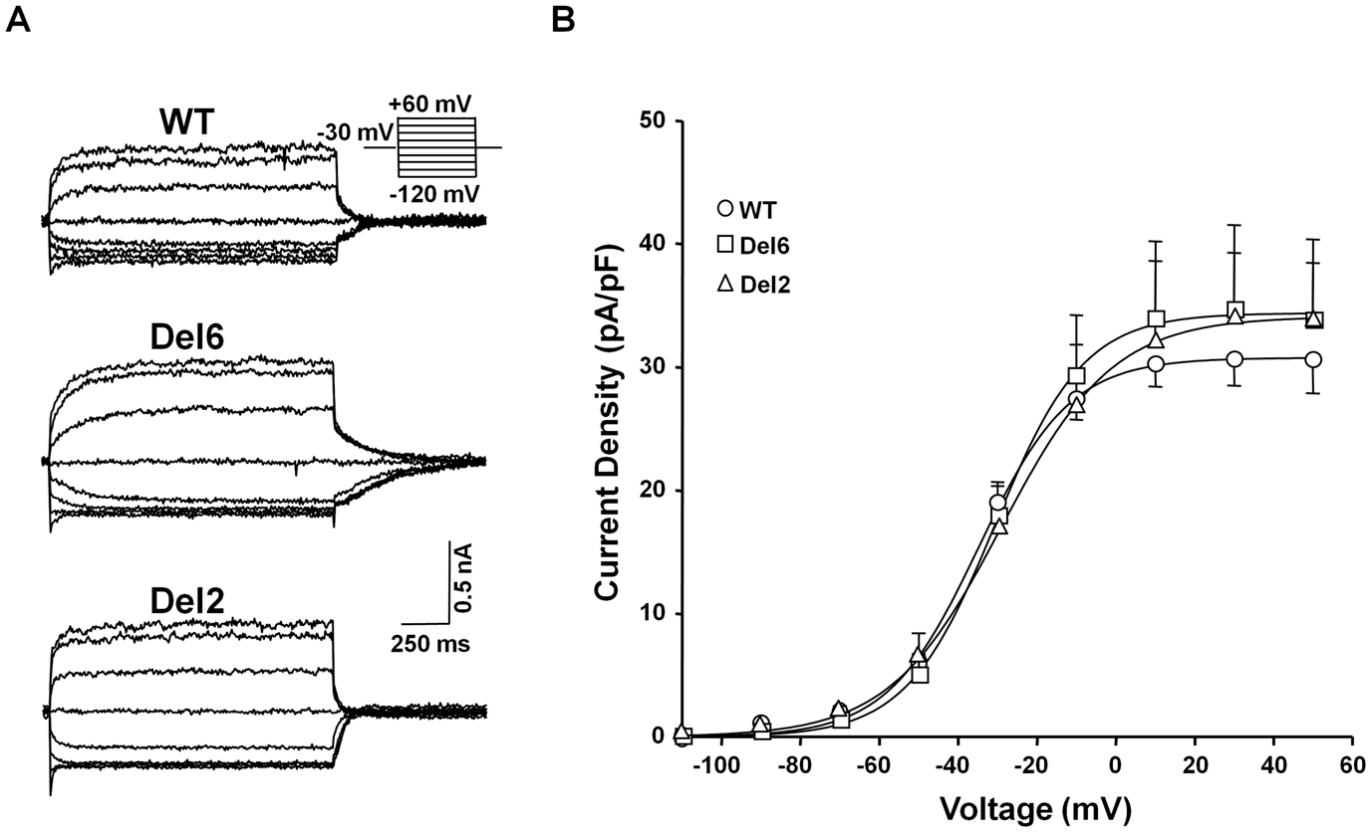
Removal of the A-B linker resulted in functional Kv7.2 channels. *A.-* Representative currents recording from HEK293T cells transfected with WT-, Del6- or Del2-Kv7.2, activated from a holding potential (V_h_) = −30 mV after 1,500 ms steps to the indicated voltages. ***B.-*** Current density-voltage relationship from tail currents of WT (*n* = 13) or Del6 (*n* = 15) channels. Each point represents the mean ± SEM. A Boltzmann equation D = Dmax/(1+e^((V-V^_1/2_^)/S)^) was fitted to the data. The averaged Boltzmann parameters were: WT: V_1/2_ =  −34.8±1.9 mV, Slope = 11.6±1.7, Dmax = 30.7±0.9 pA/pF; Del6: V_1/2_ =  −30.6±5.1 mV, Slope = 11.2±4.5, Dmax = 34.4±2.6 pA/pF; Del2: V_1/2_ =  −29.6±5.5 mV, Slope = 14.2±4.5, Dmax = 34.1±2.6 pA/pF.

Removing the linker between helices A-B is compatible with channel function (Fig. 4). HEK293T cells expressing mutant subunits yielded functional channels. In this set of experiments, we took advantage of the presence of a fluorescent tag on the subunits to select cells with similar level of protein expression. Thus, differences of the current size cannot be attributed to changes in the total number of channels in the cell. Nevertheless, the averaged current density tended to be higher in Del6 expressing cells, although due to the data scattering, the difference did not reach statistical significance. Similarly, the electrophysiological behavior of the cells expressing the Del2 mutant subunit was indistinguishable from that of the cells expressing the WT subunits. On the other hand, the impact of the deletions on the Boltzmann parameters was not significant (see Fig. 4 legend). Notably, we found that there was no correlation between subunit expression -based on fluorescent signal- and functional electrophysiological detection.

**Figure 5:**
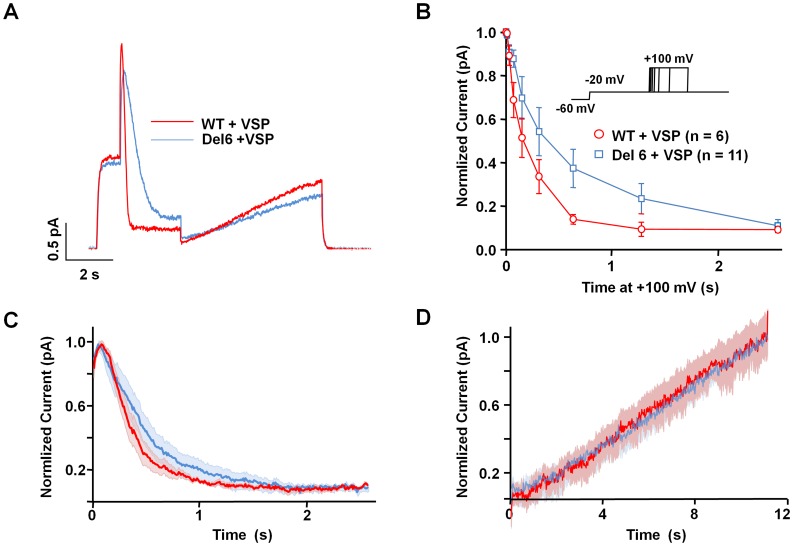
Removal of the A-B linker did not decrease PIP_2_ affinity. *A.-* Current recorded in cells transfected with WT and *Danio rerio* voltage dependent phosphatase (VSP, red) and Del6+ VSP (blue) activated from a holding potential (V_h_) = −60 mV. The initial pulse to −20 mV opens the channels without activating VSP. The second pulse to +100 mV opens additional channels faster than VSP is activated, giving rise to an initial current increase, followed by a decline. The decline phase is governed by the reduction on PIP_2_ levels at a rate that depends on the PIP_2_ affinity of the channels. After a variable period (2.560 ms in this example), voltage is returned to −20 mV, and the relative inhibition can be measured. This protocol is described in detail in [40]. ***B.-*** Averaged time course of the current decline during 2.560 ms VSP activation at +100 mV in cells transfected with WT + VSP (red; *n* = 6) and Del6+ VSP (blue; *n* = 11) subunits. The shadows represent the mean ± SEM. ***C.-*** Normalized current at −20 mV (after/before step to +100 mV) for different durations at +100 mV. Each point represents the mean ± SEM for 6–11 cells. The data from cells expressing Del6 subunits presented a large scatter, and the differences with the data from WT expressing cells did not reach statistical significance. ***D.-*** Time-course during recovery at −20 mV. The shadows represent the mean ± SEM for data from cells expressing WT (red) or Del6 (blue) channels. The rate of recovery after PIP_2_ depletion was indistinguishable.

The PIP_2_ sensitivity is not reduced after A-B loop removal. It has been shown that the loop between helices A and B has a strong influence on PIP_2_ dependent function of Kv7.2 subunits [27]. The current view is that PIP_2_ binding is essential for Kv7 channel function. Due to the PIP_2_ dependence, it can be expected that disruption of the proposed PIP_2_ site will lead to a large reduction in functional channels. To test this hypothesis, we evaluated the PIP_2_ sensitivity of the channels using a voltage-dependent phosphatase. The advantage of using this phosphatase is that no other signaling pathways, such as Ca^2+^ or PKC activation, are recruited, simplifying the interpretation of the results. In the experiment shown in Fig. 5, a current relaxation is evoked by a depolarization step to −20 mV, a voltage at which the phophatase is not activated. This control pulse reveals the basal current levels. The second pulse to +100 mV activates the phosphatase. In response to this second pulse more channels open, as revealed by the initial current increase. Simultaneously, the levels of PIP_2_ decrease, causing, after a delay, a reduction on the current. Providing that the rate of PIP_2_ resynthesis is the same, as shown in Fig. 5D, the rate of current reduction is related to the affinity for PIP_2_, such as the faster the rate, the lower the affinity (Fig. 5B). In addition, the final current levels depend on the duration of the test pulse (Fig. 5C), and from the relation between pulse duration and current levels, the relative affinity can be evaluated. We found that the rate of current reduction was slower in Del6 channels, but the difference was not statistically significant (Fig. 5B). Consistent with this result, the relation between pulse duration and current levels also suggested that the channels without the A–B linker had a higher PIP_2_ affinity (Fig. 5C). However, the differences did not reach statistical significance due to the scatter of the data from cells expressing Del6 channels. Taking all these observations together, we can conclude that removal of the A–B linker does not cause a reduction in PIP_2_ affinity.

**Figure 6:**
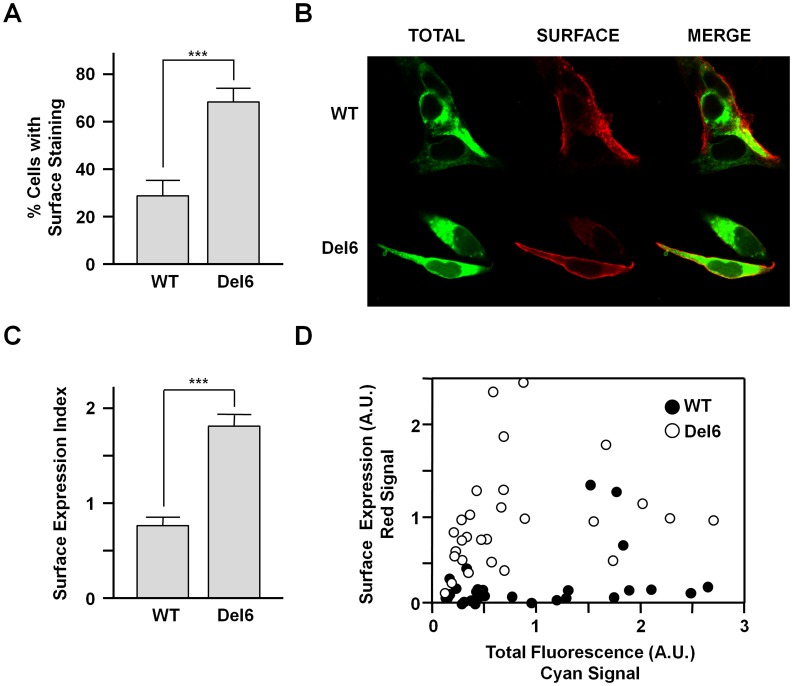
Surface expression of WT and Del6 Kv7.2 subunits in human HEK293T cells. Analysis of confocal images of non-permeabilized cells expressing the indicated constructs. The subunits have a mCFP tag at the N-terminus (rendered in green) and an extracellular 2×HA tag, allowing simultaneously monitoring total (green) and surface expression (red). The proportion of the cells with surface staining in confocal images was determined in >40 mCFP positive cells for each construct in three independent experiments. ***A.*** *-* Grey bars represent mean ± SEM of the percentage of cells expressing the channel at the surface. *** *P*≤0.001; unpaired Student’s *t* test. ***B.*** *-* Representative images of cells expressing the indicated subunit. ***C.***- Ratio of surface/total expression (red fluorescence/cyan fluorescence) from wide field epifluorescence images of cells expressing the indicated Kv7.2 subunits. *** *P*≤0.001; unpaired Student’s *t* test. ***D.***- Plot of the cyan fluorescence *vs* red fluorescence intensity from wide field epifluorescence images of cells expressing the subunits indicated. There was no correlation between total and surface expression (>90 cells from more than 5 independent experiments).

There is a significant increase in the number of cells displaying surface staining when transfected with Del6 *vs* WT subunits (Fig. 6). To examine surface expression in mammalian cells, subunits with an extracellular epitope tag were employed. Transfected non-permeabilized cells were surface immunostained and examined by epifluorescence followed by confocal microscopy. The presence of this extracellular epitope in conjunction with an N-tagged fluorescent protein allowed us to monitor surface and total expression simultaneously. Cells with comparable total fluorescence signal were selected for the analysis. Similar to the whole-cell recording experiments, the variations in surface expression cannot be ascribed to differences in total protein. Of the cells expressing WT subunits, only about 35% presented a clear surface expression signal, whereas this percentage increased to 60% in the population expressing the Del6 mutant subunit. Thus, in addition to favoring protein yield, removal of the linker also appears to facilitate the traffic of the channels to the plasma membrane. In addition, the inspection of the images revealed that a direct correlation between total protein levels and surface expression did not exist. Fig. 6B includes a striking example that illustrates this lack of correlation: The bottom of the Fig. 6B shows a field with two cells with similar protein expression levels. Whereas a robust surface signal is present in one cell, no label is discernible in the other. A similar pattern was found in >90% of the fields examined. Quantifying the total and surface expression in wide field epifluorescence images also demonstrated the increase in membrane staining after removal of the A-B linker (Fig. 6C). A plot of total *vs* surface signal from more than 90 cells for each subunit is displayed in Fig. 6D, further highlighting the absence of correspondence between these two parameters. These observations are consistent with the functional variability observed in the electrophysiological recordings described previously.

**Figure 7:**
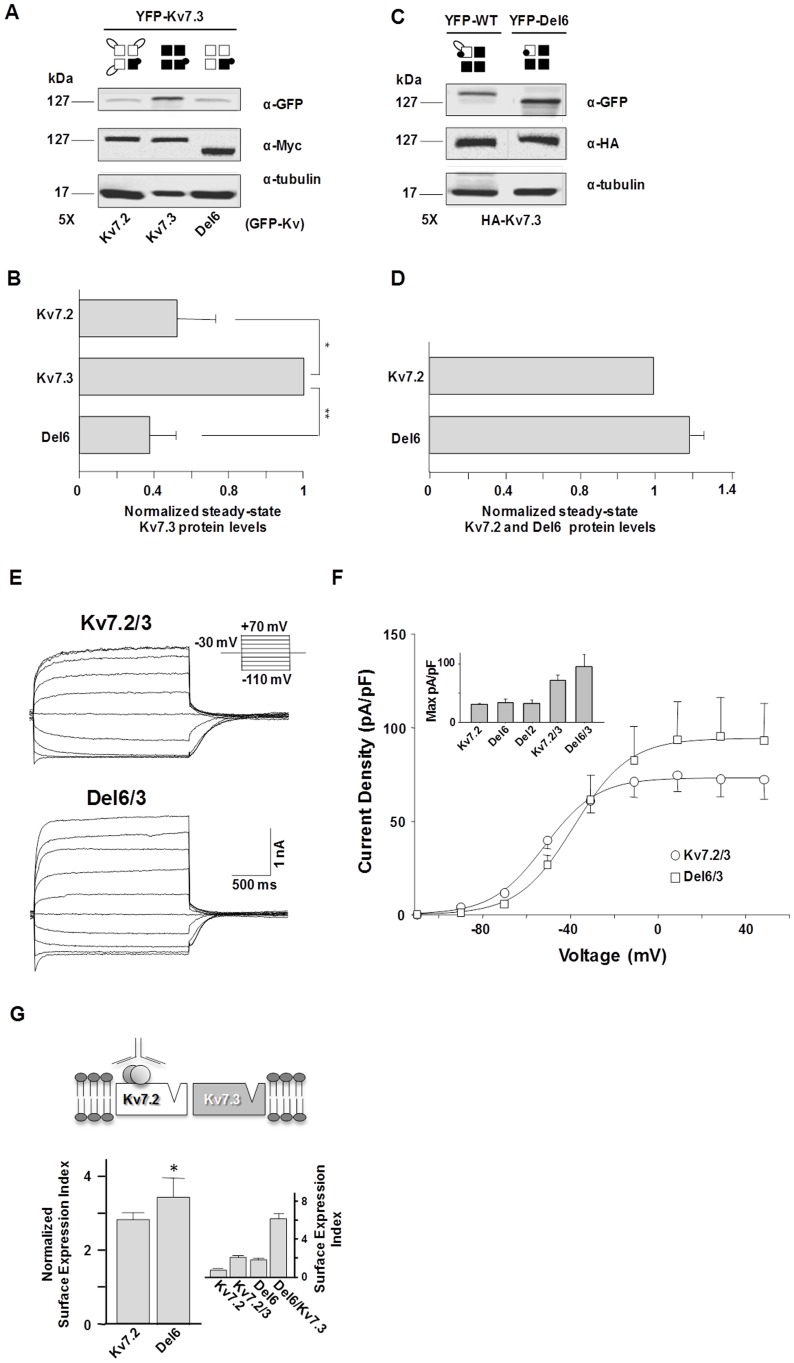
The Kv7.2 A–B linker is not critical for Kv7.2 mediated reduction on Kv7.3 protein levels. *A.-* Western blot of protein extracts from HEK293T cells transfected with YFP-Kv7.3 and the constructs indicated at the bottom of each column in a 1∶5 ratio. The constructs indicated at the bottom did not have a fluorescent protein tag, and were not detected by the anti-GFP antibody. ***B.*** *-* Densitometric quantification of the band intensity relative to the tubulin signal, normalized to the value obtained for cells overexpressing Kv7.3. Overexpression of WT- or Del6-Kv7.2 caused a significant reduction in the signal of YFP-Kv7.3, and the extent reduction was not statistically different. Bars represent mean ± SEM from 5 experiments. ** *P*≤0.01; * *P*≤0.05; paired Student’s *t* test. ***C.***- Western blot of protein extracts from cells expressing WT or Del6 subunits tagged with YFP (detected with the anti GFP antibody) in conjunction with Kv7.3 (tagged with HA) in a 1∶5 ratio. ***D.***- Densitometric quantification as in B from the data obtained as in C from three independent experiments. No significant differences were observed on the relative abundance of WT or Del6 subunits upon Kv7.3 overexpression**.** ***E.***- Representative current recording from HEK293T cells transfected with Kv7.3 and WT- or Del6-Kv7.2, activated from a holding potential (V_h_) = −30 mV after 1,500 ms steps to the indicated voltages. ***F.-*** Current density-voltage relationship from tail currents of Kv7.3 co-expressed with WT or Del6 (*n* = 7) Kv7.2 subunits. Each point represents the mean ± SEM. The averaged Boltzmann parameters were: Kv7.2/3: V_1/2_ =  −38.2±8.5 mV, Slope = 12.6±5.1, Dmax = 73.0±3.4 pA/pF; Del6/3: V_1/2_ =  - ±3.5 mV, Slope = 12.0±3.1, Dmax = 94.3±8.4 pA/pF. Inset: Maximal current density from cells expressing the indicated subunits (data from Fig. 4D and 6D). * *P*≤0.05 for maximal density; unpaired Student’s *t* test. ***G.*** Influence of Kv7.3 in surface expression of Kv7.2 WT and Del6 subunits. On top, cartoon representing the scheme of the experiment, indicating that only Kv7.2 subunits are detected at the surface due to the presence of an extracellular tag. Middle, bar graph of the surface expression index normalized to the same index obtained without Kv7.3 (see inset and Fig. 5C). The relative increase in surface expression caused by Kv7.3 was significantly larger for Del6 subunits. * *P*≤0.05; unpaired Student’s *t* test.

The Kv7.2 A–B linker does not have a major impact on the steady-state protein level of Kv7.3 subunits. Kv7.3 subunits are a main component of the M-current, and facilitate surface expression and function of Kv7.2 subunits. To test whether the Kv7.2 A–B loop affected Kv7.3 protein yield, WT or Del6 Kv7.2 were co-expressed with Kv7.3 subunits in a 5∶1 ratio, and the intensity of protein bands were examined by Western blot. The Kv7.3 subunits were tagged with YFP, and revealed with anti-GFP. For reference, YFP-tagged Kv7.3 subunits were co-expressed with molar excess of Kv7.3 subunits without the YFP tag, which are not detected with the anti-GFP antibody. Fig. 7 shows that Kv7.2 subunits caused a significant reduction on Kv7.3 protein signal. However, the impact on Kv7.3 protein yield of WT and Del6 was indistinguishable (Fig. 7B). Similarly, the protein levels of WT and Del6 channels were not affected by overexpression of Kv7.3 subunits (Figs. 7C and D). The functional impact of the linker on the heteromers was examined and, as previously shown in oocytes [10], [11], HEK293T cells expressing heteromeric channels displayed larger currents (inset in Fig. 7E) and greater surface expression (Fig. 7F). In accordance with the results on protein abundance, the linker had no significant influence on the current density, although there was a tendency for Del6/Kv7.3 heteromers to yield larger current (Fig. 7F). However, Kv7.3 had a significant influence on the surface expression of Kv7.2, and while the surface expression Kv7.2 WT almost tripled, that of Del6 subunits nearly quadrupled (Fig. 7G). Taken together, these data indicate that removal of the linker favors the surface expression of Kv7.2/3 heteromers.

## Discussion

Our data clearly show that the linker connecting helices A and B is not essential for channel function. Instead, the cells that express linker-less subunits yield more protein and allow the flow of similar current intensity than that of cells expressing WT subunits. These data suggest that the linker does not have a major impact on the maximal current (or the multiplication of the number of channels times the maximal probability of the channels being open) attainable, but it does almost double the probability that the channel reaches the plasma membrane.

We can infer that, if the currents are similar but there are twice as many channels at the membrane, removal of the linker does indeed reduce the function of the channels. The basis of this reduction is unknown, but clearly is not due to a reduction on PIP_2_ affinity. In addition to PIP_2_, other regulators such as PKC or CaM converge in the CaM binding site causing a reduction in channel activity [28]. Presumably, removal of the linker causes a restructuration of the CaM binding domain leading to a diminution on the probability of channel opening.

The rules that govern traffic to the membrane of Kv7 channels are poorly understood. The pore, the assembly domain and CaM have been shown to play important roles in trafficking [9]–[11], [13], [19]. The surface expression analysis on human HEK293T cells revealed a remarkable lack of correlation between total and surface expression. In other words, an increase in protein production does not necessarily lead to an increase in surface expression and function. This lack of correlation between protein amount and function indicates the existence of unknown underlying variables within the cell that play a major role on trafficking. What is the nature of these variables? Given its implication on trafficking, CaM is a likely candidate. However, in preliminary experiments overexpressing CaM we still observe a great degree of variability, suggesting that factors other than CaM are involved. Although CaM binds to helices A-B, we have found that removal of the linker has a small impact on the interaction and, based on the analysis of a series of CaM-binding perturbing mutants, the *in vitro* results would predict a reduction in surface expression [19]. Thus, the results of the binding assays suggest that CaM is not playing an important role in the linker-mediated effects.

We have found that the protein yield depends on the number of loops present in the channel, such that cells expressing channels with four loops have much lower steady-state level than cells expressing loop-less channels. What is the mechanism for this loop-dependent decrease in protein production? The simplest explanation for the observed results is that the loop promotes the degradation of the protein, and the more loops are present the faster the degradation rate. However, we did not obtain any evidence supporting this view. On the contrary, the degradation rate of loop-less subunits appeared to be slightly faster than that of channels with four loops. If the degradation rate is not increased, we are immediately forced to propose that the synthesis rate is reduced as a function of the number of the loops present in the tetrameric assembly.

Little is known regarding protein rate synthesis regulation in eukaryotes. It is well documented that nascent peptide-dependent translation arrest is crucial for the quality control of eukaryotic gene expression. Along the ribosomal exit tunnel, pausing due to charge-specific interactions between the tunnel and nascent peptide has been inferred [29]. Although *in vitro* elongation is a relatively uniform process, some heterogeneity has been observed *in vivo*, leading to the proposal that elongation of nascent chains may be the target of biological regulation [30]–[33]. Because the loops in one Kv7 subunit affect the yield of a different subunit, the lack of effect on the degradation rate implies that the synthesis of the subunits may be coupled. In other words, if the rate of synthesis of one subunit is very slow (because it has a loop) that will cause slowing down the synthesis of another subunits. How can this be? One possibility is that the subunits assemble before the synthesis is completed so the loop in one subunit affects the fate of the whole complex. In fact, studies carried out with Kv1.3 potassium channels, which have an N-terminal tetramerization domain called T1, have demonstrated that T1 tetramers form between neighboring subunits while the nascent channel peptides are still attached to ribosomes, and perhaps before the monomer is completely synthesized [34]. In Kv7 channels, the A-B loop is located after the transmembrane domain, which in some channels is sufficient for tetramerization. The fact that the effect of the loop is subunit specific suggests that helices C and D, which have been implicated in Kv7 subunit specific assembly [12], [35], may play a role on the process. However, these two helices are located downstream the A-B linker. This proposal implies that the loop will influence the rate of synthesis even after it has been fully read by the ribosome. It has been proposed that channels with a C-terminal assembly domain may be held in the translocon and released into the bilayer in a coupled event with C-terminal tetramerization [36]. However, the temporal and spatial sequence of events for assembly and translocation of Kv7 channels is completely unknown.

Another puzzling observation is that the Kv7.2 loop did not affect the production of Kv7.3 subunits, and the presence of Kv7.3 did not affect the relative total protein levels of Kv7.2 with and without the loop. What is the basis for this specificity? One possibility is that the synthesis rate of Kv7.3/Kv7.2 is so slow that further delays caused by the Kv7.2 loop are inconsequential. Alternatively, the loop may function in conjunction with other determinants that are not present in Kv7.3, or there are other regions on Kv7.3 that neutralize the effect of the loop. By differentially affecting the synthesis rate of homomers and heteromers, the loop will favor the production of one species over the other. This differential effect has potentially important physiological consequences. Co-expression of Kv7.2 and Kv7.3 leads to greater surface expression, and the generation of much larger currents than when either subunit is expressed alone [11], [35], [37]. Additionally, channels produced following Kv7.2/Kv7.3 co-expression have ionic permeation and conductive properties distinct from those produced by individual expression of Kv7.2 or Kv7.3 [38]. Neurons contain a diverse repertoire of ion channel complexes, and the cell must ensure that associating subunits coexist spatially and temporally. The differential effect of the A-B loop on Kv7.2 homomers and Kv7.2/3 heteromers could therefore influence the formation of different combinations of subunits, probably favoring the formations of heteromers.

## Materials and Methods

### Ethics Statement

Surgical removal of ovary tissue from adult *Xenopus laevis* females followed protocols approved under regulation 1201/2005 of Ministerio de Agricultura, Pesca y Alimentación. This study has been specifically approved by the Comité de ética de bienestar animal (CEBA/AOEB) at Universidad del País Vasco with authorization *CEBA/8/2009/VILLARROEL MUÑOZ./Extracción de ovocitos de Xenopus/*. Animals were anesthetized by immersion in water containing 0.2% w/v tricaine (MS-222, Sigma) for 5 min, and subsequently placed on ice during surgical treatment. All efforts were made to minimize animal suffering.

### Molecular Biology

The human Kv7.2 (Y15065) and Kv7.3 (NM004519) cDNAs were provided by T. Jentsch (Leibniz-Institut für Molekulare Pharmakologie, Berlin, Germany). Subunits tagged at the N-terminal with mCFP (celurean, for Kv7.2) or mYFP (citrine, for Kv7.3) were cloned into pCDNA3.1, these N-terminal tags having no impact on the electrophysiological properties of the channel [6], [39]. Where indicated, the Kv7.2 subunit was tagged at the N terminus with a tandem repeat of five Myc epitopes (MEQKLISEEDLN) and the Kv7.3 subunit was tagged with a tandem repeat of two HA epitopes (YPYDVPDYA) and cloned into pSRC5. Dr-VSP-IRES-GFP (Dr-VSP) from zebrafish (*Danio rerio*) was provided by Y. Okamura (Osaka University, Osaka, Japan). Nedd4-2 in pcDNA3.1 was provided by Cecilia Canessa (Yale University, New Heaven, USA).

### Cell Culture and Transfection

HEK293T cells (HEK 293T/17, ATCC, CRL-11268) were maintained at 37°C and 5% CO_2_ in Dulbecco’s Modified Eagle’s Medium (DMEM, Sigma-Aldrich), supplemented with non-essential amino acids (Sigma) and 10% FBS (Lonza). Cells were transiently transfected with cDNAs using a calcium phosphate protocol.

*Antibodies -* The following primary monoclonal antibodies were used: mouse anti-Myc (1∶1,000; 9E10, Sigma-Aldrich); rat anti-HA (1∶1,000; 3F10; Roche Applied Science); mouse anti-CaM (1∶2,000; 05–173, Millipore); mouse anti-GFP (1∶2,000; clones 7.1 and 13.1; 1814460, Roche Applied Science); mouse anti-tubulin (1∶3,000; DM1A, Sigma-Aldrich). The secondary antibodies used were a peroxidase-coupled anti-mouse IgG (1∶5,000; 1706516, Bio-Rad Laboratories), anti-rat IgG (1∶5,000; SC20321, Santa Cruz) and a fluorescent secondary goat anti-rat AlexaFluor 594 (1∶1,000; A11007, Invitrogen). Proteins were visualized using SuperSignal West Pico Chemiluminiscent Substrate (34078, Pierce) and SuperSignal ELISA Femto (37075, Pierce). At least 10 cumulative images (30 s exposition) were acquired using the Versadoc Imaging System (Bio-Rad Laboratories). Protein bands were analyzed with ImageJ software v1.45.

### Drug Treatments

Transfected HEK293T cells grown in T25 flasks were treated with cycloheximide (CHX, 75 µg/mL, C4859, Sigma) for distinct times and then they were analyzed by Western blotting. For lysosomal inhibition, cells were treated 10 h with 50 µM chloroquine (C6628, Sigma), and with 20 µM MG132 (474790, Calbiochem) for proteosomal inhibition [6].

### Pulse and Chase

Approximately 1.7×10^6^ cells were cultured in a T75 flask for 24 h before they were transfected with cDNA encoding the mCelurean N-tagged subunits using the lipofectamine protocol. The culture was split in four T25 flasks each corresponding to a chase time (0, 1, 2 and 4 hours). Cells were rinsed twice and starved with 3 ml of depletion medium Met- and Cys-free DMEM (D0422, Sigma), 1% FBS, 20 mM Hepes (15630-049, Gibco), 4 mM Glutamine (G8540, Sigma) for 1 h at 37°C. Following starvation, cells were labeled for 1 h at 37°C with 2 ml of Labeling medium (L-DMEM) containing 50 µlCi/ml methionine/cisteine [^35^S] (MP Biomedicals). The radioactive medium was then removed and the cells were maintained 0 to 4 h with complete DMEM containing 10% FBS until harvest. For immunoprecipitation (IP) studies with mouse anti-GFP antibodies, the cells were lysed in IP buffer containing (in mM) 50 Tris-HCl, 150 NaCl, 2 EDTA, 5 EGTA, 1% TritonX100 and protease inhibitor cocktail (1× Complete; 11836145001, Roche). The nuclei were pelleted at 500×*g* for 3 min, followed by centrifugation at 15,000×*g* for 10 min to remove the insoluble material. Lysates were incubated with Protein A-Ab for 4 h at 4°C and, after two washes with IP buffer, immunoprecipitated proteins recovered after heating at 90°C for 5 min in SDS sample buffer.

### Electrophysiological Measurements

HEK393T cells were used for whole-cell patch recordings, which were obtained at RT (21–25°C) 48 h after transfection using lipofectamine 2000 (Invitrogen). Cells were bath perfused with the following solution (mM): 140 NaCl, 4 KCl, 2 CaCl_2_, 2 MgCl_2_, 10 HEPES (Na), 5 D-glucose, adjusted to pH 7.4 with NaOH. The osmorality was adjusted with mannitol to ∼315 mOsm. Pipettes were pulled from borosilicate glass capillaries (Sutter Instruments, USA) using a Narishige micro-pipette puller (PC-10; Narishige Instrument Co., Japan). Membrane currents were measured using an EPC-8 amplifier (HEKA Instruments, Germany) with pipette and membrane capacitance cancellation. The composition of the solutions for the experiments testing the effect of VSP was taken from [40]. For the external medium was (in mM) 160 NaCl, 2.5 KCl, 2 CaCl_2_, 1 MgCl_2_, 10 HEPES and 8 glucose, pH 7.4 (NaOH) and the internal solution was 175 KCl, 5 MgCl_2_, 5 HEPES, 0.1 K_4_BAPTA, 3 Na_2_ATP, pH 7.4 (KOH). Pipettes were filled with an internal solution containing (mM): 125 KCl, 10 HEPES (K), 5 MgCl_2_, 5 EGTA, 5 Na_2_ATP, adjusted to pH 7.2 with KOH and the osmolarity adjusted to ∼300 mOsm with mannitol. The access resistance was typically 2–3 MΩ. The amplitude of the Kv7 current was defined as the peak difference in current relaxation measured at −30 mV after 1.500 ms pulses to −120 mV (all channels closed) and to +60 mV (all channels opened). The data were acquired and analyzed using pCLAMP software (version 8.2), normalized in Excel (Microsoft Corp., Madrid, Spain) and plotted using SigmaPlot (SPSS Corp., Madrid, Spain). Data are shown as the mean ± SEM (*n* indicates the number of samples). The differences between the means were evaluated using the unpaired Student’s *t* test, with values of *P*≤0.05 considered significant.

### Surface Expression and Confocal Microscopy

Mutant Kv7.2 subunits tagged with an HA epitope in the extracellular loop connecting the S1 and S2 transmembrane domains were used in chemiluminescent assays of individual oocytes from female *Xenopus laevis* [10], [11]. To increase accessibility, the epitope was flanked with fragments from the extracellular D1–D2 loop of the ClC-5 chloride channel [10]. The background signal from uninjected oocytes was subtracted (background represented <10% of the maximal signal). In addition, confocal microscopy was used to analyze surface expression of the full-length channels in non-permeabilized cells. The subunits were tagged at the N-terminus with mCelurean (CFP), and they contained a HA tandem between S1 and S2, modifying the sequence between transmembrane domains S1 and S2 to ^115^KEYEKSSEGSEH**YPYDVPDYA**G**YPYDVPDYA**VTFEERDKCPEWNA^126^. We confirmed that the expression of these S1–S2 tagged subunits did not affect the typical macroscopic currents [10]. HEK293T cells grown on coverslips coated with 1 mg/mL poly-L-Lysine (P1524, Sigma) were washed with PBS 36 h after calcium phosphate transfection. The cells were fixed with freshly diluted 3% paraformaldehyde in PBS for 20 min, washed three times with PBS and then pre-incubated for 30 min with 5% BSA. The primary antibody (anti-HA diluted 1∶1,000 in blocking solution) was then added for 1 h, after which the cells were washed three times in 5% BSA in PBS and exposed for 1 h to the secondary antibody (AlexaFluor 594-conjugated goat anti-rat IgG, Invitrogen) diluted 1∶1,000 in blocking solution. For epifluorescence experiments (Figs. 5C, D; 6E), the preparation was incubated for 45 min with a tertiary antibody (AlexaFluor 594-conjugated donkey anti-goat IgG, Invitrogen) diluted 1∶1,000 in blocking solution. The cells were then washed three times in PBS and mounted with ProLong Antifade reagent (P36930, Invitrogen). Images from stained cells were captured using a 60×, NA 1.45 oil objective on a Nikon Eclipse TE2000-U fluorescence microscope equipped with a confocal module using a 30 µm pinhole. ImageJ software (Version WCIF, National Institutes of Health, USA) was used to quantify surface expression. Fluorophores were excited with a 408 nm laser (Coherent) for CFP, or a 560 nm laser line (Melles-Griot) for AlexaFluor 594. The emission filters used were BA 485/40 (Chroma) for CFP and BA593/40 (Nikon) for AlexaFluor 594. Epifluorescence images were captured using a 40×, NA 1.3 oil objective. For YFP the filters were Excitation: D495/10X (Chroma), Dichroic: 515CDLP (Chroma), Dichroic: 525LP (Nikon); Celurean using Semrock filter CFP set, Excitation: FF01-438/24-25, Emission: FF01-483/32-25, Dichroic: FF458-Di01-25×36); and Alexa594 using Semrock red set (Excitation: FF01-562/40-25, Dichroic: FF01-624/40-25, Dichroic: FF593Di02-25×36).

### Recombinant Protein Production and Fluorescence Analysis

CaM dansylation (D-CaM), purification of CaM and GST-fusion proteins (Kv7.2 CaM-binding site, G310-L548), and fluorescent analysis was performed as described previously [19], [39].

## Supporting Information

Figure S1 Shown is a degradation assay using a proteasome inhibitor (20 µM MG132) and a lysosome inhibitor (50 µM chloroquine; CQ) in HEK293T cells. Cells expressing the indicated subunits were treated with inhibitors for 10 h. Samples were loaded in lanes from the left in the following order: control without any treatment (24 h), vehicle (PBS) for the CQ treatment and DMSO for the MG132 treatment. After the 10 h incubation periods, the protein extracts were separated by SDS-PAGE and analyzed by Western blotting using anti-Myc and anti-tubulin antibodies. Similar results were obtained in three independent experiments.
(TIF)
