# Supplementary figures and images for: Surface Expression and Subunit Specific Control of Steady Protein Levels by the Kv7.2 Helix A-B Linker
Source: PLoS One. 2012 Oct 24;7(10):e47263. doi: 10.1371/journal.pone.0047263 (PMC3480381; doi:10.1371/journal.pone.0047263)

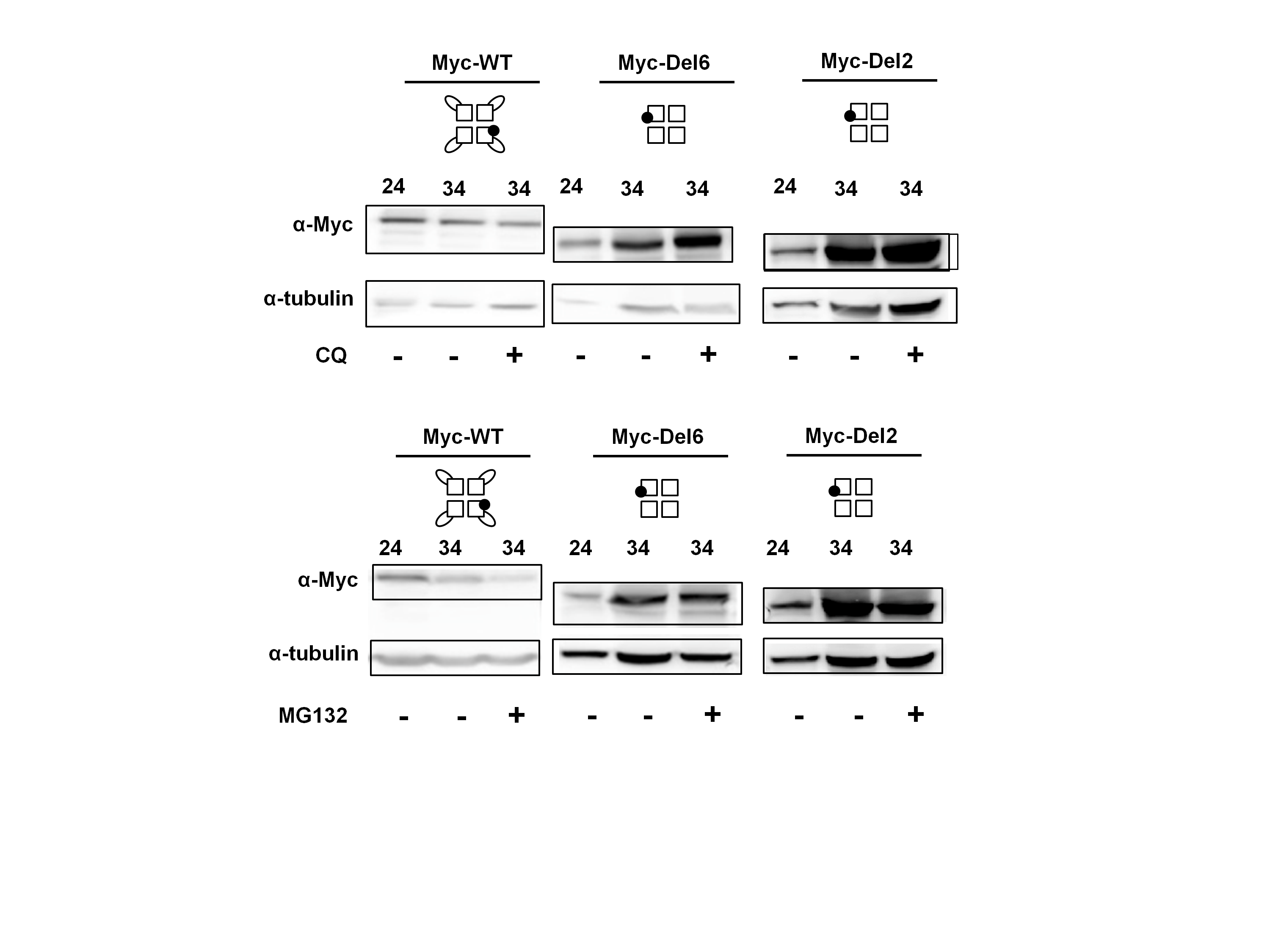

Supplement: Figure S1 — Shown is a degradation assay using a proteasome inhibitor (20 µM MG132) and a lysosome inhibitor (50 µM chloroquine; CQ) in HEK293T cells. Cells expressing the indicated subunits were treated with inhibitors for 10 h. Samples were loaded in lanes from the left in the following order: control without any treatment (24 h), vehicle (PBS) for the CQ treatment and DMSO for the MG132 treatment. After the 10 h incubation periods, the protein extracts were separated by SDS-PAGE and analyzed by Western blotting using anti-Myc and anti-tubulin antibodies. Similar results were obtained in three independent experiments. (TIF) [file pone.0047263.s001.tif]
